# Chemical Sensing and Chemoresponsive Pumping with Conical-Pore Polymeric Membranes

**DOI:** 10.3390/nano10030571

**Published:** 2020-03-21

**Authors:** Stevie N. Bush, Thomas T. Volta, Charles R. Martin

**Affiliations:** Department of Chemistry, University of Florida, Gainesville, FL 32611, USA; stevie.walters@chem.ufl.edu (S.N.B.); tgaliber@chem.ufl.edu (T.T.V.)

**Keywords:** ion-current rectification, electroosmotic flow rectification, chemoresponsive pump, conical pore membranes, sensors, electroosmotic pump

## Abstract

Synthetic membranes containing asymmetrically shaped pores have been shown to rectify the ionic current flowing through the membrane. Ion-current rectification means that such membranes produce nonlinear current–voltage curves analogous to those observed with solid-state diode rectifiers. In order to observe this ion-current rectification phenomenon, the asymmetrically shaped pores must have pore-wall surface charge. Pore-wall surface charge also allows for electroosmotic flow (EOF) to occur through the membrane. We have shown that, because ion-current is rectified, EOF is likewise rectified in such membranes. This means that flow through the membrane depends on the polarity of the voltage applied across the membrane, one polarity producing a higher, and the opposite producing a lower, flow rate. As is reviewed here, these ion-current and EOF rectification phenomena are being used to develop new sensing technologies. Results obtained from an ion-current-based sensor for hydrophobic cations are reviewed. In addition, ion-current and EOF rectification can be combined to make a new type of device—a chemoresponsive nanofluidic pump. This is a pump that either turns flow on or turns flow off, when a specific chemical species is detected. Results from a prototype Pb^2+^ chemoresponsive pump are also reviewed here.

## 1. Introduction

Synthetic membranes containing pores that are asymmetrically shaped have been of considerable recent research interest [1,2,3,4,5,6,7,8]. Examples include pyramidally shaped pores in mica sheets [9,10] and conically shaped pores in polymeric membranes [11]. Membranes containing such pores have been used in sensor development, such as resistive pulse sensors for DNA [12], and in the development of a new type of electroosmotic flow (EOF) based pump [11,13]. This review discusses recent advances in the use of conical pore polymeric membranes in sensor development and EOF pumping. Of particular interest is very recent work that combines these sensing and pumping concepts to make a new device, a “chemoresponsive” pump [13]; i.e., a pump that turns on or off in response to a specific chemical stimulus. 

Both the sensing and pumping applications make use of a phenomenon observed with asymmetric-pore membranes called ion-current rectification (ICR) [11,14]. The theory of ICR has been discussed in detail [8,15,16,17,18] and is not reviewed in this paper. Instead, this review describes how conical-pore polymeric membranes are prepared, and then moves on to applications of such membranes in chemical sensing. We then review electroosmotic flow in such membranes, with special emphasis on driving EOF with alternating current (AC), as opposed to the more typical direct-current-based EOF. The advantages of AC EOF are reviewed, and finally, very recent work that combines both the sensing and pumping technologies into a single device is discussed. This has led to a new type of device, a “chemoresponsive” pump [13]; *i.e.*, a pump that turns on or off in response to a specific chemical stimulus.

## 2. Conical Pore Membrane Fabrication

Conical pore membranes are prepared by using the well-known track-etch method [19,20]. This process involves first irradiating a sheet of a polymer with energetic heavy ions from a nuclear reactor or cyclotron, to create latent tracks through the polymer. The tracked membrane is then chemically etched to convert the latent tracks into pores. Filtration membranes of this type with cylindrical pores have been available commercially for decades [21,22]. Polymers used to make such track-etched membranes include polyethylene terephthalate (PET) [23,24], polycarbonate (PC) [25], and polyimide (PI) [26]. 

**Figure 1:**
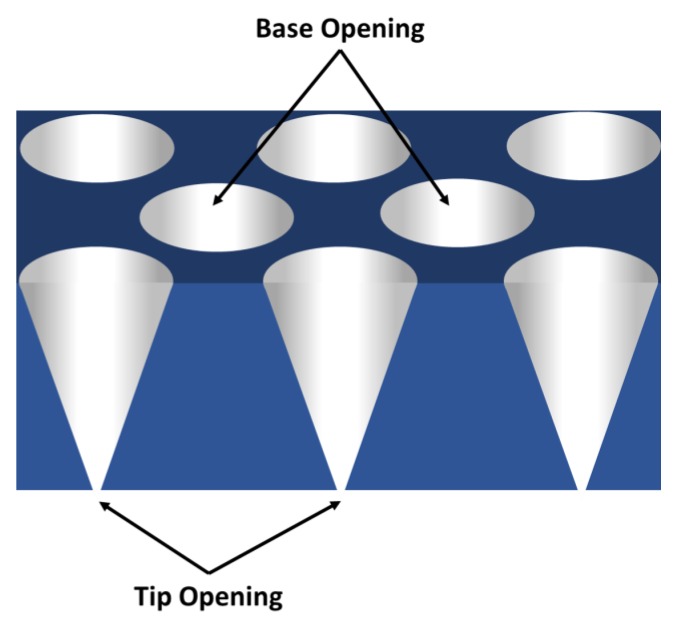
Schematic illustration of a membrane containing conical pores. Dimensions are not drawn to scale.

To make the conventional cylindrically shaped pores, the tracked membrane is simply immersed into the etching solution [21,22]. However, fabrication of conically shaped pores requires the membrane to be mounted between two halves of a U-tube cell [18]. A solution that etches the polymer is placed in one half-cell, and a solution that neutralizes the etchant is placed in the other half-cell. The latent track starts etching from the face exposed to the etch solution. Ultimately, the etch solution breaks through to the stop solution, to give a conically shaped pore, with the larger base diameter exposed to the etchant, and the smaller tip diameter exposed to the stop solution (Figure 1). 

**Figure 2:**
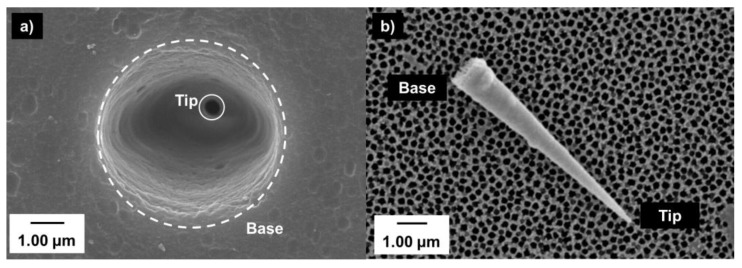
SEM of (**a**) base opening in a track-etched conical-pore polycarbonate membrane; (**b**) a gold replica of a conical polyethylene terephthalate pore.

While etching, a constant voltage is applied between two Pt or Au wires placed with the anode in the etching solution and the cathode in the stopping solution. Before breakthrough, the current through the membrane is essentially zero. Breakthrough is signaled by the sudden flow in current through the now-continuous pores. The membrane can then be etched further, if needed, and the etching time is controlled to reach the desired tip and base diameters. If the same etching conditions are used, then base diameters of the track-etched nanopores will be reproducible [27]. The diameter of the pore bases can be determined from scanning electron micrographs (SEM), like that shown in Figure 2, or by using an electrochemical method described previously [20,28,29]. 

This one-step etching process creates variability in tip diameters. Thus, a second etching process can be used to combat this issue [27]. After the first etching step is completed to the desired conical nanopore base size, the membrane is exposed to an isotropic chemical etching process, where the entire length of the nanopore is etched uniformly, with the same etchant placed on both sides of the membrane [27]. Again, a constant voltage is applied between two wires placed in the two etching solutions. The magnitude of current that flows through the conical nanopores is uniquely related to the conical nanopore tip diameter [29]. Therefore, by etching until a predetermined current value (*i.e.*, a predetermined tip size) is obtained, the variability in the conical nanopore tip diameters is removed [27]. Pores with base diameters of 420 nm to 1.4 μm and tip diameters of 22 to 67 nm were used for the sensing and pumping applications reviewed here [11,13,26]. 

Figure 2a presents a SEM of the surface of a PC conical-pore membrane, with the base opening of the pore facing up, and the tip facing down. The internal pore structure can be imaged by plating correspondingly conically shaped gold wires in the pores [30]. The polymeric membrane can then be dissolved away [31] and the gold wires, which are replicas of the pores, are collected by filtration and imaged (Figure 2b). These gold structures can also be used to confirm the formation of conical nanopores [32]. Further information on the track-etch process is available in the literature [33]. 

## 3. Ion-Current and Electroosmotic Flow Rectification in Conical Pore Membranes

The theories of ion-current rectification (ICR) and electroosmotic flow rectification (EFR) have been discussed in detail and are not reviewed here [18]. Briefly, to observe these phenomena in a porous membrane, the pores must be asymmetrically shaped (Figure 1 and Figure 2), and the pore walls must have excess surface charge [8,15]. The track-etched polymeric membranes described here have excess negative charge due to the presence of surface carboxylate groups that results from the etching process [34]. PET membranes have a surface charge density of ~12 mC per m^2^ [23,24], while PC has a value of ~2 mC per m^2^ [25].

**Figure 3:**
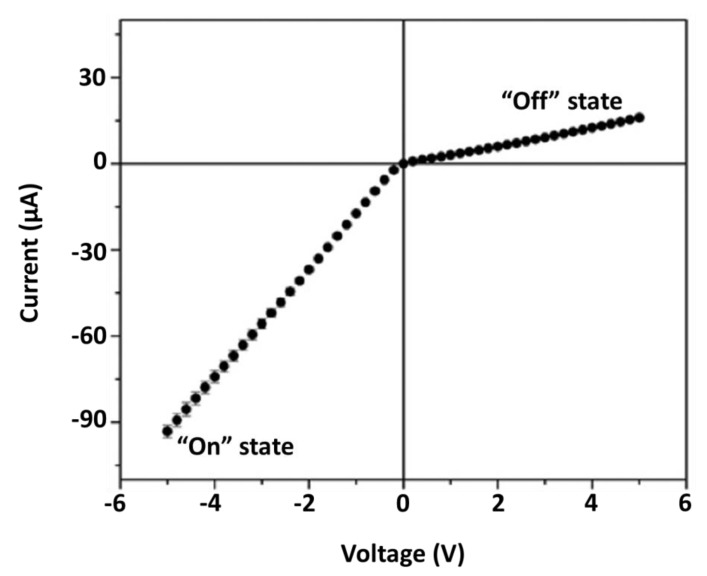
Rectified current–voltage (I–V) curve in 25 mM KCl buffered with 1 mM Na_2_HPO_4_, measured across a PET membrane with conical pores. Reprinted and adapted with permission from [11]. Copyright 2016, American Chemical Society.

To observe ICR and EFR, the conical-pore membrane is mounted in a U-tube cell, and an electrolyte solution is placed in each half-cell [18,26]. An electrode immersed into each solution is used to apply a voltage ramp across the membrane and measure the resulting ionic current flowing through the pores. For pores with tip diameters of 100 nm or less [35,36,37,38,39], the resulting current–voltage (I–V) curve is nonlinear. Specifically, the slope of the I–V curve for one sign of applied transmembrane voltage is lower than for voltages with the opposite sign (Figure 3). In analogy to a solid-state diode rectifier, the lower slope observed at positive values of transmembrane voltage in Figure 3 is called the “off” state, and the higher slope region at negative values of transmembrane voltage is the “on” state [15,40]. 

It is well-known that electroosmotic flow can be driven through porous membranes that have excess charge on the pore walls [8,15]. As per the measurement of ion-current (Figure 3), EOF is often accomplished in a U-tube cell with an electrolyte solution in each half-cell. Again, an electrode in each half-cell is used to pass an ionic current through the pores. For electrolyte concentrations of less than ~25 mM, fluid flow can be observed through the membrane. If the surface charge is negative, EOF will be in the direction from the anode half-cell to the cathode half-cell. That is, EOF is in the direction of the migrating counter cations associated with the fixed negative surface charge on the pore wall [18].

Because the rate of EOF through the membrane is dependent on the magnitude of the current flowing through the pores, we reasoned that, in a conical-pore membrane, EOF should be rectified just as the ionic current is rectified [9,41]. This means that the rate of fluid flow through the conical pore membrane is lower for one sign of applied transmembrane voltage than for voltages of the opposite sign. Higher fluid flow rates are observed at voltages corresponding to the “off” state (Figure 3), and lower flow is observed for voltages corresponding to the “on” state. This means that the flow is higher in the direction base-opening to tip-opening and lower in the direction tip-to-base [41]. A simple way to remember this is that, in the high flow condition, the fluid is following in the direction of flow through a funnel, base-to-tip. 

The extent of ICR can be quantified by the rectification ratio, *r_ic_* [35,36,37,38,39]. Assuming the membrane has fixed negative surface charge, *r_ic_* is defined by the current measured at a specified value of negative applied transmembrane voltage divided by the current at the same value of positive voltage [42]. The corresponding EFR rectification ratio, *r_eof_*, is calculated from the ratio of the higher EOF velocity, base-to-tip, over the lower velocity, tip-to-base. A key conclusion from our prior work is that the EOF rectification ratio, *r_eof_*, increases with increasing ion-current rectification ratio, *r_ic_* [35,36,37,38,39]. This is because both phenomena derive from the presence of the excess negative surface charge, and both entail electrical migration of the counter cations associated with that charge. The sensing and chemoresponsive pumping applications reviewed next also derive from the excess surface charge. The key to these applications is the ability to chemically change the quantity of that charge.

## 4. Ion-Current Rectification-Based Sensing 

As noted above, to observe ion-current rectification, the membrane must contain asymmetrically shaped pores with excess pore-wall surface charge. We have shown that, if the surface charge can be removed, for example, by protonation of the surface carboxylate groups, ICR is no longer observed [43]. In the sensing application reviewed here, adsorption of an analyte ion removes the surface charge. As a result, the extent of rectification varies with the concentration of the analyte ion, with r_ic_ decreasing with increasing analyte concentration [26,43]. 

**Figure 4:**
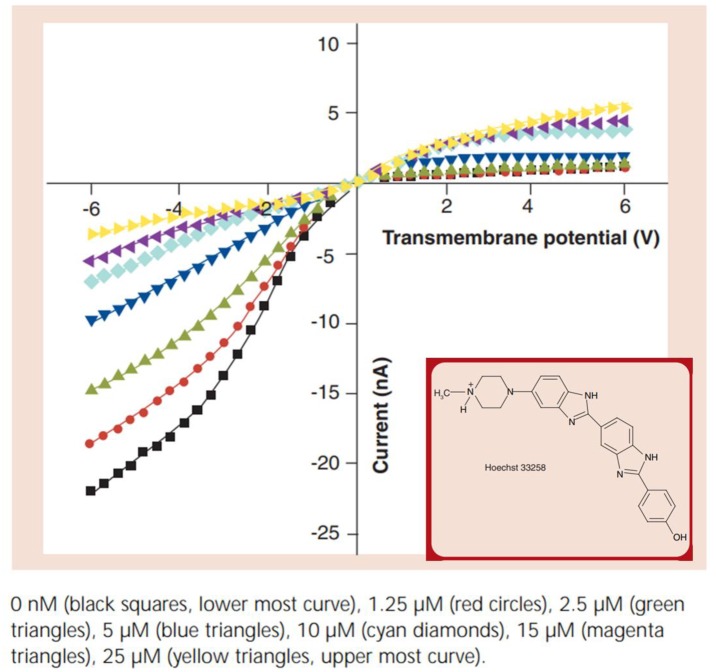
Rectified I–V curve in the presence of various concentrations of Hoechst 33258. Reprinted and adapted with permission from [26]. Copyright 2008, Future Medicine.

Figure 4 shows data for a polyimide membrane (12 μm thick) containing a single asymmetrical pore. Membranes with two different base and tip diameters were used with an average base diameter of 1.7 ± 0.4 μm and a tip diameter of 60 ± 10 nm. The analyte in this case is a cationic yet hydrophobic molecule, Hoechst 33258 (inset, Figure 4). In the absence of Hoechst 33258, ICR is observed with the “on” state occurring at negative potentials and the “off” state at positive applied potentials [26]. As noted above, this indicates that the PI membrane has negative surface charge [15,40]. As shown in Figure 4, when the membrane is exposed to Hoechst 33258, the extent of rectification decreases. This is because hydrophobic polymers with fixed negative surface charge show high ion-exchange affinities for hydrophobic cations [44]. As a result, the hydrophobic Hoechst 33258 molecule adsorbs to the pore wall, effectively neutralizing the excess negative surface charge [26].

As the concentration of the Hoechst 33258 increases, the extent of rectification, as quantified by *r_ic_*, decreases, and *r_ic_* scales inversely with the concentration of Hoechst 33258 (Table 1). Eventually, at high analyte concentrations, ICR is reversed, signifying a change in the sign of the pore-wall surface charge. This means that the net surface charge is now positive, indicating that the quantity of adsorbed hydrophobic cation exceeds the quantity of the fixed negative surface charge. 

**Figure 5:**
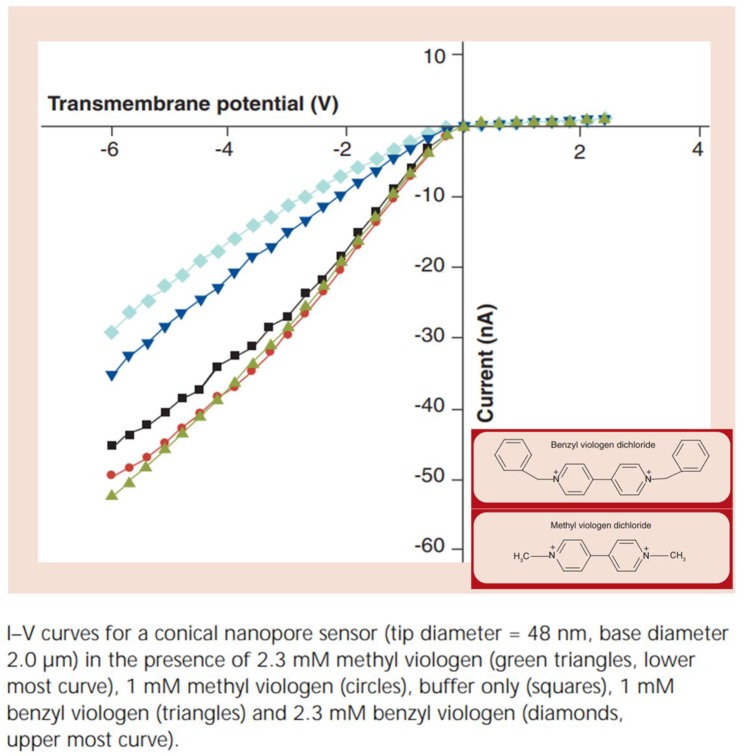
Rectified I–V curve in the presence of various concentrations of methyl viologen and benzyl viologen. Reprinted and adapted with permission from [26]. Copyright 2008, Future Medicine.

To show that the hydrophobic nature of the analyte cation is responsible for the change in *r_ic_* observed (Figure 4; Table 1), two organic divalent cations, methyl viologen and benzyl viologen, were studied. At the pH used, Hoechst 33258 is a monovalent cation [45], whereas the viologens are divalent [26]. If the interaction of the analyte ion with the pore surface was dominated by electrostatics, it would be predicted that methyl viologen (MV^2+^) would have the strongest interaction with the surface. This is because, out of these three cations, MV^2+^ has the highest charge-to-molecular-mass ratio. The data show, however, that MV^2+^ has no measurable interaction with the surface, because the current–voltage curves obtained in the presence of MV^2+^ are within experimental error identical to those obtained in the buffer solution without an organic cation (Figure 5).

In contrast benzyl viologen (BV^2+^) causes the extent of ICR to decrease, indicating that this more hydrophobic divalent organic cation has a stronger interaction with the surface than MV^2+^. These BV^2+^ vs. MV^2+^ data reinforce the conclusion that hydrophobic interactions dominate the ion/surface interaction for a charged hydrophobic surface [44]. This is again reinforced by the BV^2+^ vs. Hoechst 33258 data. The most hydrophobic Hoechst 33258 has the strongest surface interaction because the concentration of BV^2+^ required to produce a quantifiable change in ICR is orders of magnitude higher than that of Hoechst 33258 [26]. 

## 5. Alternating Current EOF Pump

EOF is of practical importance because it is commonly used to pump fluids through microfluidic devices and capillary electrophoresis columns [18]. As discussed above, asymmetric pore membranes that exhibit ion-current rectification also show the related phenomenon electroosmotic flow rectification. We recently showed how to develop a practical pumping technology based on the rectified EOF phenomenon [11,18]. This was accomplished by using a sinusoidal voltage waveform to drive EOF through the pores in a PET membrane [11,46,47,48]. 

**Figure 6:**
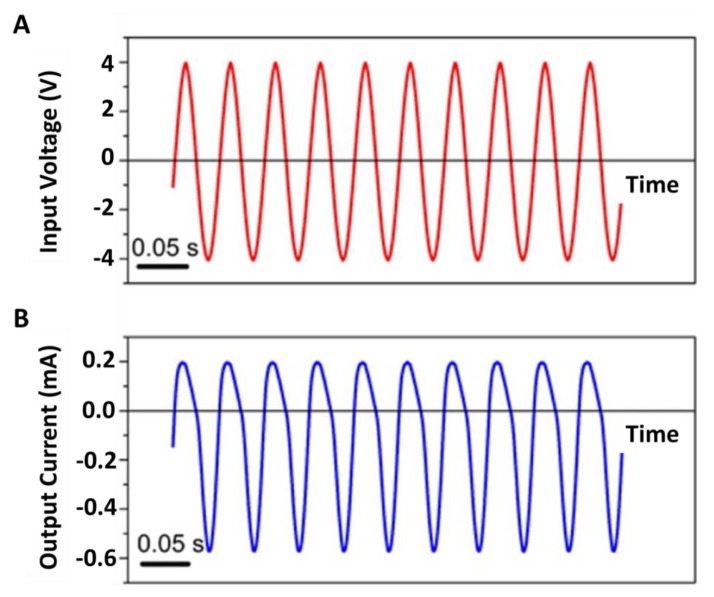
(**A**) Applied sinusoidal voltage waveform with magnitude of 2.8 *V_rms_* and frequency of 20 Hz. (**B**) Resulting ion current. Reprinted and adapted with permission from [11]. Copyright 2016, American Chemical Society.

When such a symmetrical sinusoidal voltage waveform is applied across the membrane (Figure 6a), the resulting alternating current (AC) flowing through the membrane is also rectified (Figure 6b). This causes the flow through the membrane to be likewise rectified, with flow in the direction base-to-tip higher than flow in the opposite direction. Therefore, a net flow through the membrane in the direction base-to-tip is observed when the symmetrical sinusoidal voltage waveform is applied [11]. We call this the AC EOF pump.

**Figure 7:**
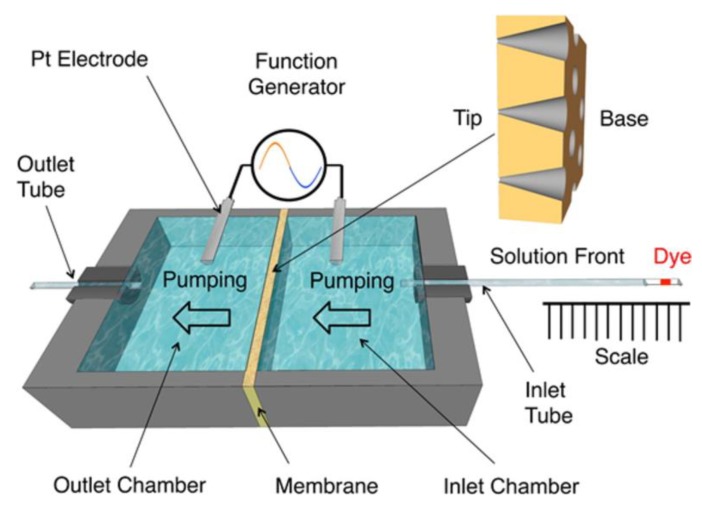
Schematic illustration of the AC electroosmotic flow (EOF) pump cell. A dye plug in the inlet tube was used to determine the EOF velocity. A net flow from base-to-tip was observed. Reprinted with permission from [11]. Copyright 2016, American Chemical Society.

A schematic of the cell used to demonstrate AC EOF pumping is shown in Figure 7. A conical pore PET membrane (12 μm thick and 10^7^ pores/cm^2^) with base and tip diameters of approximately 420 ± 30 nm and 22 ± 2 nm, respectively, was mounted between an inlet and an outlet chamber, with the base opening facing the inlet chamber [11]. The sinusoidal voltage waveform was applied between two Pt electrodes that were placed in each chamber. 

**Figure 8:**
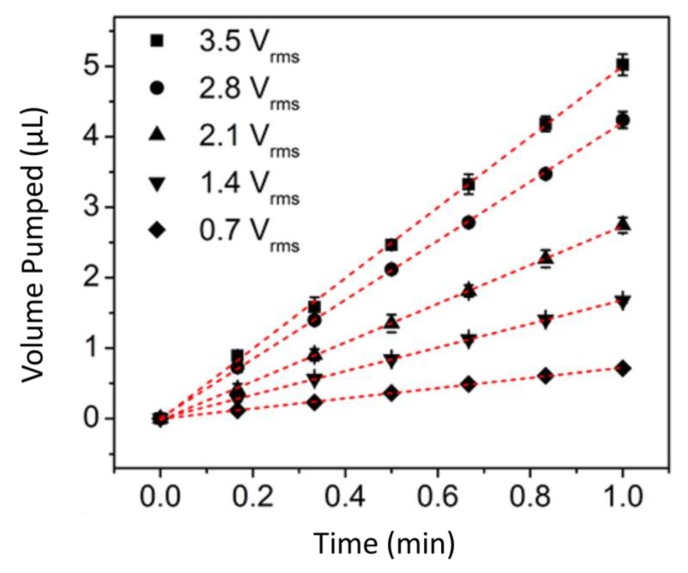
Volume pumped vs. time as the indicated values of V_rms_ at a frequency of 20 Hz. Reprinted and adapted with permission from [11]. Copyright 2016, American Chemical Society.

The EOF flow rate was determined by measuring the rate of movement of a plug of dye solution through the inlet tube sealed to the inlet chamber (Figure 7). These data were used to calculate the volume of solution pumped vs. time (Figure 8). At all values of *V_rms_* used, the volume pumped was found to increase linearly with time, indicating a constant flow rate through the membrane. Since the slopes of the lines in Figure 8 are the flow rates, these data also show that flow rate increases with increasing sinusoidal voltage amplitude, *V_rms_* [11]. 

EOF is typically driven at constant current or constant voltage. This might be called DC EOF, as opposed to the AC EOF described here. When EOF is driven in this conventional DC way, water is reduced at the cathode of the device, and water is oxidized at the anode. Since it takes energy to split water, driving EOF in these traditional DC modes wastes energy. A key advantage of the AC EOF pump is that water-splitting is not necessary. As discussed in detail in [11,18], this is because double layer charging currents, as opposed to the Faradaic current causing water splitting, dominate in the AC experiment. 

**Figure 9:**
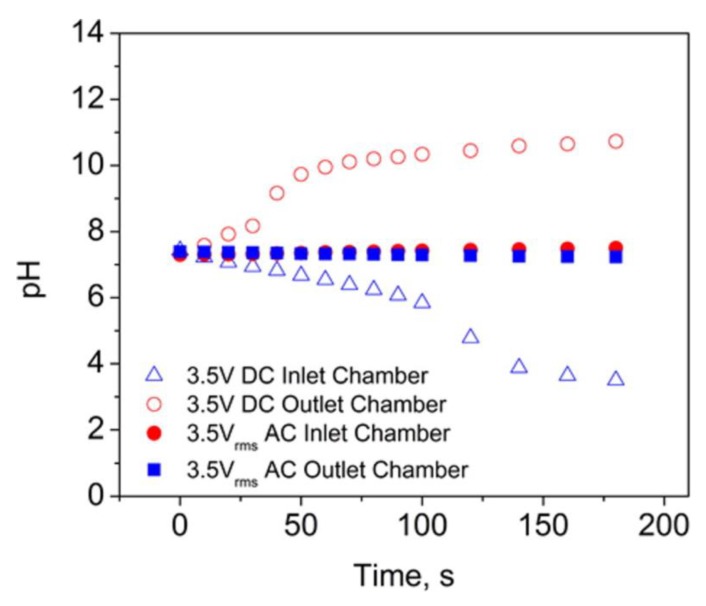
pH of the inlet and outlet chambers vs. time while driving EOF with 3.5 V DC and 3.5 *V_rms_* AC. Reprinted with permission from [11]. Copyright 2016, American Chemical Society.

In addition to wasting energy, water electrolysis occurring at the electrodes in DC mode produces H_2_ gas bubbles at the cathode and O_2_ gas bubbles at the anode. This is undesirable because the bubbles generated can block the flow [46,47,48]. Again, since double-layer charging currents dominate in AC mode, water electrolysis and concomitant bubble generation are suppressed. This was proven by monitoring the pH of the inlet and outlet chambers while flow was being driven through the membrane (Figure 9). In DC mode, the pH of the outlet chamber increased with time due to water reduction, whereas the pH of the inlet chamber decreased due to water oxidation [11]. Alternatively, in AC mode, there was no change in pH of either solution over the time window investigated.

## 6. Chemoresponsive Nanofluidic Pump

We recently combined the ion-current rectification sensing technology (Section 4) with the AC EOF pumping technology (Section 5), to make a “chemoresponsive pump” [13]. To our knowledge, this was the first report of such a device—a pump that either turns flow on or off in response to a specific chemical signal. Conceptually, a chemoresponsive pump combines both sensing and pumping functions into a single device [13]. 

In our prototype device, Pb^2+^ was the chemical species detected, and flow was turned off in the presence of sufficient concentrations of this specific analyte ion. This was accomplished in a manner analogous to the cationic drug sensor reviewed above, in that, in both cases, the anionic surface charge on the pore wall was neutralized by the cationic analyte. In the drug-sensor case, this occurred by adsorption of a hydrophobic analyte cation to the hydrophobic pore wall. In the case of Pb^2+^, a chemically selective ionophore, 18-crown-6 [11,46,49], was covalently attached to the pore walls. Moreover, 18-crown-6 has a high binding selectivity for Pb^2+^, with a formation constant of 1.86 × 10^4^ [50,51]. It shows much lower affinity for common inorganic cations, such as Na^+^ and K^+^ [50,51]. Since 18-crown-6 is electrically neutral, the complex formed with Pb^2+^ is positively charged, and in this way, the anionic surface charge on the pore wall is neutralized.

**Figure 10:**
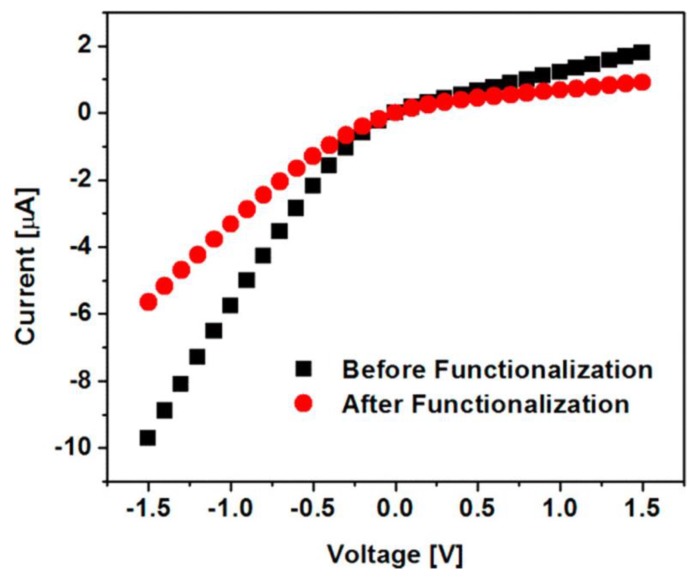
I–V curves for conical pore membranes before and after pore-wall functionalization with 18-crown-6. The electrolyte in both the receiver and feed solution was 10 mM LiCl and 5 mM Tris-HCl (pH 7.0). Reprinted with permission from [13]. Copyright 2018, American Chemical Society.

Well-known EDC chemistry was used to attach an amino-functionalized 18-crown-6 to a fraction of the carboxylate sites on the pore walls [13]. Since attachment in this way forms a neutral amide bond, the anionic surface charge density is lowered upon binding of the 18-crown-6. As a result, the ion-current rectification ratio was lower after functionalization (Figure 10). Only a fraction of the carboxylate sites is functionalized due to size constraints. The 18-crown-6 molecule has a diameter of about 1.2 nm [52], while the average distance between carboxylate groups on the pore wall is ~1 nm [53]. Because the pore walls remain negatively charged, an AC EOF pump based on the 18-crown-6 functionalized membrane is “turned on” in the absence of Pb^2+^. 

**Figure 11:**
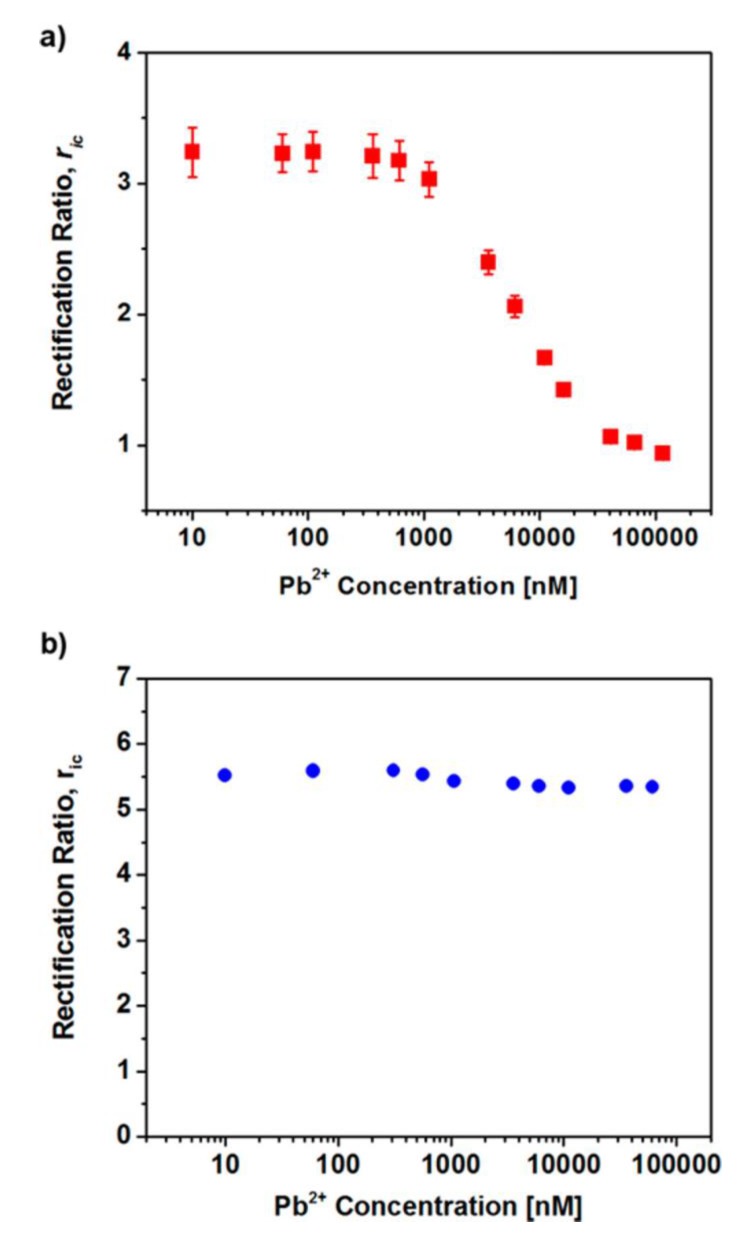
(**a**) Ion-current rectification ratio as a function of Pb^2+^ concentration for an 18-crown-6-functionalized PET membrane. (**b**) Analogous plot for a membrane with no attached 18-crown-6. Reprinted with permission from [13]. Copyright 2018, American Chemical Society.

To prove that Pb^2+^ was bound to the 18-crown-6-functionalized pore-walls, the ion-current rectification ratio was measured as a function of the concentration of Pb^2+^ in contact with the membrane [13]. A plot of *r_ic_* versus Pb^2+^ concentration (Figure 11a) shows that, at a concentration above ~1 μM, the extent of ICR decreased with increasing Pb^2+^ concentration. Again, this is because, as Pb^2+^ binds to the ionophore, it effectively titrates away the excess negative surface charge density [13]. No change in ion-current rectification is observed for a control membrane with no attached ionophore (Figure 11b).

**Figure 12:**
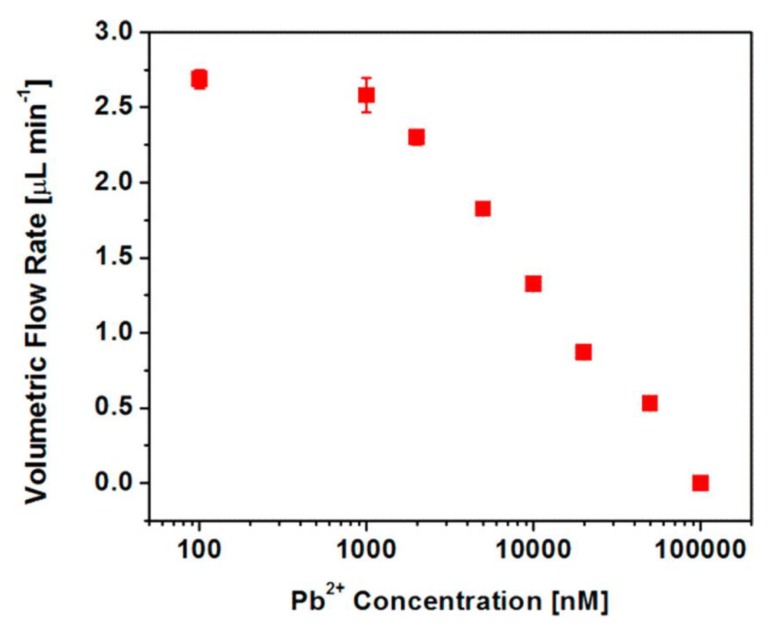
Volumetric solution flow rate through the functionalized conical pore membrane as a function of Pb^2+^ concentration. Reprinted with permission from [13]. Copyright 2018, American Chemical Society.

Figure 12 shows a corresponding plot of EOF volumetric flow rate through the membrane as a function of Pb^2+^ concentration. In the low concentration region, below ~1 μM, where the rectification ratio has its highest (and constant) value (Figure 11a), a high flow rate is observed through the membrane. However, as per the rectification ratio data, concentrations of Pb^2+^ above ~1 μM cause flow rate to decrease with increasing Pb^2+^ concentration. This reinforces a core concept in EOF that the excess surface charge is responsible for EOF, and if that charge can be neutralized, EOF will not be observed. Both the rectification ratio data (Figure 11a) and the flow rate data (Figure 12) show that complete neutralization of the surface charge, as well as zero flow rate, is observed at Pb^2+^ concentrations above 100 μM. 

After exposure to Pb^2+^, the chemoresponsive pump can be regenerated by contacting the membrane with a solution of the chelating agent ethylenediaminetetraacetic acid (EDTA) [13]. The EDTA/Pb^2+^ complex has a formation constant of 10^18^ [54], which is about fourteen orders of magnitude larger than the 18-crown-6 complex [55]. The large difference in the formation constants causes EDTA to strip the Pb^2+^ from the 18-crown-6 on the pore wall. This restores the excess negative surface charge, which allows the pump to resume flow [13].

Finally, to prove that the pump was selective to Pb^2+^, analogous experiments were conducted with other cations. Table 2 shows the percent change in flow rate before and after exposure to 10 μM solutions of the cations studied. The formation constant for the Pb^2+^/18-crown-6 complex is at least two orders of magnitude greater than the formation constants for the other cations studied [55]. As a result, little change in flow rate is observed in the presence of these other cations. An analogy can be drawn to the organic cation sensing data reviewed above (Table 1; Figure 5). In both the pumping and sensing experiments, the device selectively responds to the cation that binds most strongly to the surface.

## 7. Conclusions

As reviewed here, conical-pore PET and PI membranes can be used as both sensing and pumping devices. Ion-current rectification (ICR), and the fact that EOF rectification scales with ICR, provides the underlying basis for these sensing and pumping functions. Furthermore, the sensing and pumping functions can be combined to make a new type of device—a chemoresponsive pump. Results from a prototype Pb^2+^ chemoresponsive pump were reviewed here.

Looking to the future, our prototype chemoresponsive pump turned the flow off when the membrane was exposed to sufficiently high concentrations of the chemical stimulus Pb^2+^. It would be just as interesting and important to develop a pump that is initially turned off (no flow), but turns on when the specific chemical stimulus is detected. This could be accomplished by first removing all the native excess negative charge on the pore walls, and then attaching a neutral binding agent for a cationic or anionic analyte. Exposure to the analyte ion would then charge the pore wall, and flow would be turned on.

Another issue that must be further addressed is selectivity. In the case of the Hoechst 33258 sensor, selectivity was based on hydrophobic interactions between this hydrophobic cationic analyte and the hydrophobic, but negatively charged, pore walls. While this form of selectivity allowed for discrimination against the less hydrophobic organic cations MV^2+^ and BV^2+^, a cation of comparable hydrophobicity to Hoechst 33258 would strongly interfere. In analogy to the Pb^2+^ chemoresponsive pump, enhanced selectivity can, in principle, be obtained by attaching a selective binding agent for the analyte of interest to the pore walls. However, this is much easier to accomplish with inorganic ions, where selective ionophores (e.g., 18-crown-6), are available. Antibodies might provide the requisite selectivity for other types of analytes. However, because these pumping and sensing devices are based on the presence, or absence, of pore-wall charge, the native charge on the protein would present a complication.

## Figures and Tables

**Table 1:** Ion-current rectification (ICR) rectification ratios as a function of Hoechst 33258 concentration. Reprinted and adapted with permission from [26]. Copyright 2008, Future Medicine.

Concentration (μM)	*r_ic_*
0	19.6
2.5	10.8
5	4.9
10	1.9
15	1.2
25	0.67

**Table 2:** Percent volumetric solution flow rate change measured after addition of the indicated cation. Reprinted and adapted with permission from [13]. Copyright 2018, American Chemical Society.

Metal Cation	Percent Flow Rate Change
Pb^2+^	54.3 ± 0.4
K^+^	10 ± 4
Na^+^	9 ± 1
Sr^2+^	9 ± 2
Cu^2+^	8 ± 3
Ca^2+^	7 ± 3
Mg^2+^	6 ± 4
Zn^2+^	6 ± 1
Hg^2+^	5 ± 4
